# In Vitro Physiological Characterization of Strains from Transcontinental and Endemic *Morchella* Species from South-Central Chile

**DOI:** 10.3390/jof12080572

**Published:** 2026-08-01

**Authors:** Mhartyn Elso, Mauricio Sanz-Rocha, Tatiana Escobar-Hernández, Yudith Guillén, Macarena Gerding, Daniel Chávez, Ángela Machuca

**Affiliations:** 1 Departamento de Ciencias y Tecnología Vegetal, Escuela de Ciencias y Tecnologías, Universidad de Concepción, Campus Los Ángeles, Juan Antonio Coloma 0201, Los Ángeles 4451032, Chile; mhelso@udec.cl (M.E.); tf.escobarhz@gmail.com (T.E.-H.); yguillen@udec.cl (Y.G.); danielchavez@udec.cl (D.C.); 2 Departamento de Producción Vegetal, Facultad de Agronomía, Universidad de Concepción, Campus Chillán, Av. Vicente Mendez 595, Chillán 3801061, Chile; msanz2126@gmail.com (M.S.-R.); mgerding@udec.cl (M.G.)

**Keywords:** morels, *Morchella andinensis*, carbon effect, nitrogen effect, mycelial growth, sclerotia, native forests, post-fire plantations

## Abstract

True morels (*Morchella* spp.) in South America are poorly documented, despite their high gastronomic and ecological value. In this study, morel ascomata were collected from various environments in south-central Chile, and their in vitro physiological performance was characterized. The endemic species *Morchella andinensis*, along with the transcontinental *M. tridentina*, *M. eximia*, and *M. importuna*, were identified, pure isolates obtained and then cultivated under various conditions. The carbon supplements had a significant effect on mycelial performance, with mannose and pectin yielding high growth rates (~13 mm·day^−1^) and biomass production (~100 mg·plate^−1^). Carboxymethylcellulose consistently reduced growth across all strains. Depending on the strain, organic nitrogen supplements such as peptone and amino acids promoted growth more than inorganic nitrogen such as ammonium sulfate. Most strains demonstrated higher performance at pH 7.2–8.5 and 24 °C, and sclerotia formation was strongly strain-dependent. An exploratory principal component analysis (PCA) descriptively summarized the standardized physiological response profiles of the eight selected strains. In this analysis, *M. importuna* strains were associated with generally higher growth responses, while *M. tridentina* T140 and *M. eximia* E87 showed comparatively lower responses. The *M. andinensis* strains occupied intermediate positions in the multivariate space. The results indicate significant physiological differences among *Morchella* strains. This underscores the importance of strain-level evaluation as a preliminary step for future studies on strain preservation, artificial cultivation trials, and biotechnological investigations of morels in Chile.

## 1. Introduction

Species of *Morchella* (*Ascomycota*, *Morchellaceae*) are known for their morphological plasticity and wide ecological range, factors that historically complicated their classification before molecular tools were widely adopted [1,2]. Recent molecular research has delineated three clades within the genus: *Esculenta*, *Elata*, and *Rufobrunnea*, with regular taxonomic updates incorporating newly described species [3,4,5]. In Chile, early morphological assessments often led to misidentifications, including erroneous references to *M. esculenta* and *M. conica*. However, a combination of modern molecular and taxonomic approaches has clarified the occurrence of five species to date: *M. aysenina*, *M. andinensis*, *M. tridentina*, *M. eximia*, and *M. importuna*. Some species are primarily associated with native *Nothofagus* forests, whereas others are frequently found in disturbed environments, including *Pinus* sp. and *Eucalyptus* sp. forest plantations [6,7]. Among these, *M. andinensis* is of relevance because it is endemic to southern South America [6,8] and, to date, has only been recorded in association with native forest ecosystems in Chile [6]. Additionally, *M. aysenina* has only been found in the native forests of Chilean Patagonia [6,9]. Meanwhile, *M. eximia* and *M. importuna* frequently inhabit disturbed areas or forest plantations [6,7,8,10].

*Morchella* mushrooms have significant commercial value, selling for around $200 USD per kg in American and European markets [11]. Although wild harvesting remains widespread across these regions, large-scale outdoor cultivation (particularly in China) focuses on *M. sextelata*, *M. importuna*, and *M. eximia*, the species most used in artificial cultivation [11,12,13,14]. In the evaluation of multiple *Morchella* strains conducted by Yue et al. [15], substantial intraspecific variability was noted, with clear differences in growth performance and physiological responses among isolates of the same species. This underscores that diversity within species can strongly affect both cultivation outcomes and the ecological behavior and distribution of the genus *Morchella*, even under standardized laboratory conditions. Notably, most research thus far has focused on cultivated species such as *M. sextelata* and *M. importuna*, and systematic comparisons across isolates are scarce for other *Morchella* species. Additionally, the in vitro evaluation of strain development under different culture media has shown that medium composition, nutrient sources, and subculturing regimen significantly influence physiological, transcriptomic, and metabolomic responses, with clear agronomic and biological consequences [16]. While many cultivated species exhibit saprotrophic behavior [5,17], field studies suggest that some may also form ectomycorrhizal or endophytic associations in natural environments [18,19,20,21]. Moreover, several species appear closely tied to disturbed forest conditions, while others appear closely tied to native forest conditions [6,7]. Accordingly, it is essential to characterize mycelial growth and developmental traits in vitro across strains and species from different habitats to understand ecological niches and predict potential trophic interactions in situ. On the other hand, advances in knowledge of in vitro nutritional strategies make it possible to identify and select *Morchella* strains that exhibit potential for artificial cultivation [22]. Overall, studies on the factors that influence mycelial growth in vitro are essential for conserving local biodiversity and for guiding sustainable harvesting and management practices, as well as improving cultivation methods [7,18].

Although new records and new *Morchella* species, including endemic taxa, have recently been reported from disturbed and undisturbed habitats in south-central Chile [6,7,10], their mycelial cultures remain poorly characterized. In contrast to several Northern Hemisphere *Morchella* species, the physiological and cultivation-related traits of Chilean lineages remain largely unexplored. Therefore, this study identified and evaluated the in vitro performance of *Morchella* species collected from native forests, forest plantations, and urban areas. Pure isolates obtained from ascomata collected in field were subsequently evaluated. The physiological responses in terms of mycelial growth and morphological traits were compared among strains in the presence of various carbon and nitrogen supplements, as well as under different pH and temperature conditions. These data provide a physiological basis for the management and preservation of Chilean *Morchella* strain collections, including native and endemic taxa. The data also contribute to identifying preliminary traits relevant in Chilean morel strains for future cultivation screening.

## 2. Materials and Methods

### 2.1. Collection of Ascomata and Study Sites

Between August and October 2022, fresh ascomata of *Morchella* were collected from native forests, forest plantations, and urban areas in different locations in south-central Chile: Villucura (Santa Barbara), Peralillo (Quilleco), Lantaño (Chillán), Copiulemu (Florida), Santa Fe (Los Ángeles), Monte El León (Pemuco). These sites represented a diverse range of environmental conditions and levels of disturbance within the Ñuble and Biobío administrative regions, which are located between 36° and 37° S latitude (Supplementary Table S1). According to local collectors, these locations had a documented history of morel occurrence at different times of the year. Ascomata were collected individually, placed in paper bags, and transported to the laboratory for subsequent molecular identification. The ascomata were photographed in situ, and dominant surrounding vegetation and some habitat characteristics were recorded (Supplementary Table S1).

### 2.2. Sample Collection for DNA Extraction

For the molecular analyses, approximately 5 g of tissue was aseptically removed from fresh ascomata with a sterilized blade and then dehydrated in sealed plastic bags containing silica gel. Approximately 25 mg of the desiccated material was ground in liquid nitrogen using an autoclaved pestle and mortar. The pulverized samples were then re-suspended in 500 μL of genomic lysis buffer. Total DNA was extracted using the Quick g-DNA Miniprep kit (Zymo Research, Irvine, CA, USA) in accordance with the manufacturer’s instructions.

### 2.3. PCR Amplification and Sequencing

Based on previous studies of *Morchella* species [6,7], the identity and phylogenetic relationships among the morel individuals were determined by ITS sequencing. Nuclear rDNA ITS1–5.8S–ITS2 (ITS) was amplified using the primers ITS1 and ITS4. The PCR reaction mix consisted of 1 µL DNA, 0.2 µL GoTaq^®^ (Promega, Madison, WI, USA), 0.5 µL primer ITS1 (50 µM), 0.5 µL primer ITS4 (50 µM), 0.2 µL dNTPs (10 mM), 10 µL 5× buffer, 3 µL MgCl_2_ (25 mM), and 34.6 µL PCR ultra-pure water. The cycling parameters were 5 min at 95 °C, followed by 35 cycles of 94 °C for 30 s, 55 °C for 30 s, and 72 °C for 90 s, with a final hold at 72 °C for 5 min. The amplification was verified by electrophoresis in 1.0% (*w*/*v*) agarose gels stained with 1:10,000 Gel Red DNA gel stain (Biotium, Fremont, CA, USA). The Kapa™ Universal Ladder was used as a molecular marker. Electrophoresis was carried out in 1× TAE buffer (40 mM Tris-acetate, 1 mM EDTA, pH 8.0) at 75 V for 2 h. Once gene amplification was confirmed, DNA samples were sent to Macrogen (Seul, Republic of Korea) for sequencing.

### 2.4. Phylogenetic Analysis

The chromatograms were analyzed and edited using Gene Tool Lite 1.0 software (Doubletwist, Inc., Oakland, CA, USA). Model-selection and phylogenetic analyses were conducted with MEGA12. The reference sequences used for phylogenetic analyses were retrieved from GenBank. Alignments were performed with ClustalX and edited manually. The final ITS alignment comprised 553 aligned positions. Substitution-model selection was performed in MEGA12 using the Maximum Likelihood model-selection procedure. Twenty-four model combinations were compared using the Bayesian Information Criterion (BIC), and K2+I (K2P+I) was selected because it had the lowest BIC value (4545.426). The phylogenetic tree was constructed using the maximum likelihood method and the K2P+I model, with a bootstrap analysis of 1000 replicates to assess node support. Gaps and missing data were treated using partial deletion with a 95% site-coverage cutoff. The web-based BLAST (Version 2.16.0) search tool from the National Center for Biotechnology Information (NCBI) was used to find *Morchella* sequences. All specimens identified were deposited in the Fungarium of the University of Concepción, Campus Los Ángeles, Chile, under the UDEC-LAF code (Table 1).

### 2.5. Isolation of Morchella Strains

*Morchella* isolates were obtained by aseptically excising inner tissue fragments from the pileus of each fresh ascoma previously identified by molecular and phylogenetic analyses. Small fragments (approximately 2 mm) were placed on malt extract agar (MEA) and subcultured repeatedly until pure cultures were obtained. Stock cultures were maintained on 2% MEA plates at 4 °C. Prior to experimental assays, 8 mm mycelial plugs were transferred from stock plates to fresh 2% MEA and incubated at 24 °C for one week. New plugs from these actively growing cultures were then used as the inoculum for each trial. This procedure was standardized across experiments to ensure a consistent physiological age of the inoculum. Finally, all strains were preserved at 4 °C on 2% MEA plates and test tubes in the culture collection at the University of Concepción, Campus Los Ángeles, Chile.

### 2.6. Selection and Culture Conditions of Morchella Strains

From the strains obtained for each species (*n* = 3–4), an initial screening was conducted to select those for subsequent in vitro analyses. Radial growth rate (mm·day^−1^) was evaluated on complete yeast medium (CYM) at 24 °C for 10 days. Based on these results, two strains per species were selected, prioritizing those showing contrasting growth rates (fastest and slowest within each species, when differences were detected). These selected strains were subsequently used in all comparative physiological assays.

### 2.7. Effect of Carbon (C) and Nitrogen (N) Supplements, pH, and Temperature on In Vitro Growth of Morchella Strains

Complete yeast medium (CYM) was used as the basal solid medium (pH 7.0) for the different in vitro conditions tested. CYM consists of 2% agar, 2% glucose, 0.2% yeast extract, 0.2% peptone, 0.1% K_2_HPO_4_, 0.046% KH_2_PO_4_, and 0.05% MgSO_4_. To evaluate the effect of different carbon (C) and nitrogen (N) supplements on the growth of *Morchella* strains, glucose and peptone were replaced in the original CYM. In the C source assay, glucose was replaced by 2% mannose, fructose, xylose, sucrose, maltose, pectin, starch, or carboxymethylcellulose. CYM glucose-free was used as a control. For the N source assay, peptone was replaced by 0.2% organic N sources: the amino acids alanine (C_3_H_7_NO_2_) and proline (C_5_H_9_NO_2_), or inorganic sources: ammonium nitrate (NH_4_NO_3_) and ammonium sulfate ((NH_4_)_2_SO_4_). CYM peptone-free was used as a control. It should be noted that the sources of C and N that were added to the basal medium were used as supplements to the nutrients contained in the CYM in the form of yeast extract. This component was retained in these assays as a vitamin source.

For the pH assay, the medium was adjusted with NaOH or HCl solutions to target pH values of 4, 5, 7, and 8 prior to autoclaving at 121 °C for 20 min under 15 psi. After autoclaving and solidification, the pH of the solid medium was measured directly on the agar surface using a flat-tip pH electrode to verify the initial pH of each treatment before inoculation. The recorded pH values were 4.5, 5.5, 7.2, and 8.5, respectively. In the temperature assay, plates containing basal CYM adjusted to pH 7 were inoculated and incubated at 10, 15, 24, and 28 °C in darkness.

Petri dishes (90 mm) were prepared with 20 mL of medium each and inoculated in the center with a single 8 mm plug of mycelial agar obtained from stock plates. All assays were performed in triplicate and incubated for 10 days at each condition in complete darkness. Each strain × condition combination consisted of three independent Petri-dish replicates (*n* = 3). Radial growth was measured daily along four axes on each plate and averaged at each time point. Radial growth was measured in four radial directions corresponding to the two perpendicular axes drawn on the bottom of each plate. In each direction, the distance from the edge of the inoculum plug to the advancing colony margin was recorded, and the four measurements were averaged for each plate at each time point. Measurements were performed at 12-h intervals for rapidly growing strains and at 24-h intervals for slower-growing strains. Radial growth rate (mm·day^−1^) was estimated separately for each plate as the slope of the linear phase of radial extension versus time. When applicable, measurements corresponding to the initial lag phase or the final interruption of radial extension caused by the colony reaching the edge of the 90-mm Petri dish were excluded from the regression analysis. At least four consecutive measurements within the linear growth phase were used for each regression. Photographic documentation was conducted for each assay. Macroscopic mycelial features, including pigmentation and growth patterns, as well as colony margin characteristics and the development of structures such as sclerotia, were recorded during incubation. At the end of each experiment, the biomass in the plates was assessed by first dissolving the agar in a microwave, then hot filtering through filter paper and washing with hot water several times. Finally, filter paper plus biomass was dried at 60 °C for 48 h to determine the dry weight (mg/plate) [7]. Control plates were prepared with C and N supplements but without inoculation. The plates were then incubated, hot filtered, and washed according to the above-described procedure. The dry weights of the resulting residues were then used to correct the dry weight values of the mycelial biomass.

### 2.8. In Vitro Sclerotia Induction Assays

To evaluate sclerotia formation in the selected *Morchella* strains, a modification of the split-plate method described by Amir et al. [23] was employed. Petri dishes (90 mm) were prepared with two media: one half contained a nutrient-deficient CYM with reduced glucose (1%), without yeast extract, peptone, or MgSO_4_, while the other half contained standard CYM. Each plate was inoculated at the edge of the nutrient-deficient side with an 8 mm agar mycelial plug. Plates were incubated in darkness at 24 °C in triplicate. After 14 days, the sclerotial structures were visually monitored and counted in each plate through digital images using ImageJ software (version 1.53r; National Institutes of Health, Bethesda, MD, USA). These sclerotia were initially characterized as mycelial aggregates that subsequently became compact, with distinguishable boundaries from the surrounding mycelium and pigmentation [24]. Contiguous structures were counted as one unit unless clearly separated. Colony development and sclerotia were photographically monitored until day 21. Because of the high variability observed among replicates in terms of the number of sclerotia formed per plate, abundance was expressed as a range rather than an average ± SD.

### 2.9. Statistical Analysis

All statistical analyses were performed using a combination of InfoStat v.2020 (Universidad Nacional de Córdoba, Argentina) and Python 3.11 (Python Software Foundation). Two-way analyses of variance (ANOVA) were applied to evaluate the effects of strain, nutrient source (carbon or nitrogen), and factors (temperature and pH), including their interactions. The ANOVA models were computed in InfoStat with type III sums of squares, while assumptions of normality and homoscedasticity were verified through residual analysis. When significant effects were detected (*p* < 0.05), Tukey’s honestly significant difference (HSD) test was used for pairwise comparisons. For visualization and complementary multivariate analyses, the statistical environment Python was employed. A principal component analysis (PCA) was performed with the scikit-learn (v.1.4.2) package, while data handling and plotting were conducted with pandas (v.2.2.1) and matplotlib (v.3.8.0). A PCA was performed on mean values of growth rate and biomass across treatments (*n* = 3 replicates per condition), using the eight strains as observations. Before analysis, each variable was centered and scaled to unit variance using z-score standardization, because radial growth rate and biomass accumulation were expressed in different units and numerical scales. Score and loading plots were generated to descriptively explore similarities among the standardized response profiles of the eight selected strains. Given the limited number of strain-level observations relative to the number of variables, the PCA was used as an exploratory and descriptive analysis.

## 3. Results and Discussion

### 3.1. Morchella Species and Natural Habitats

**Figure 1:**
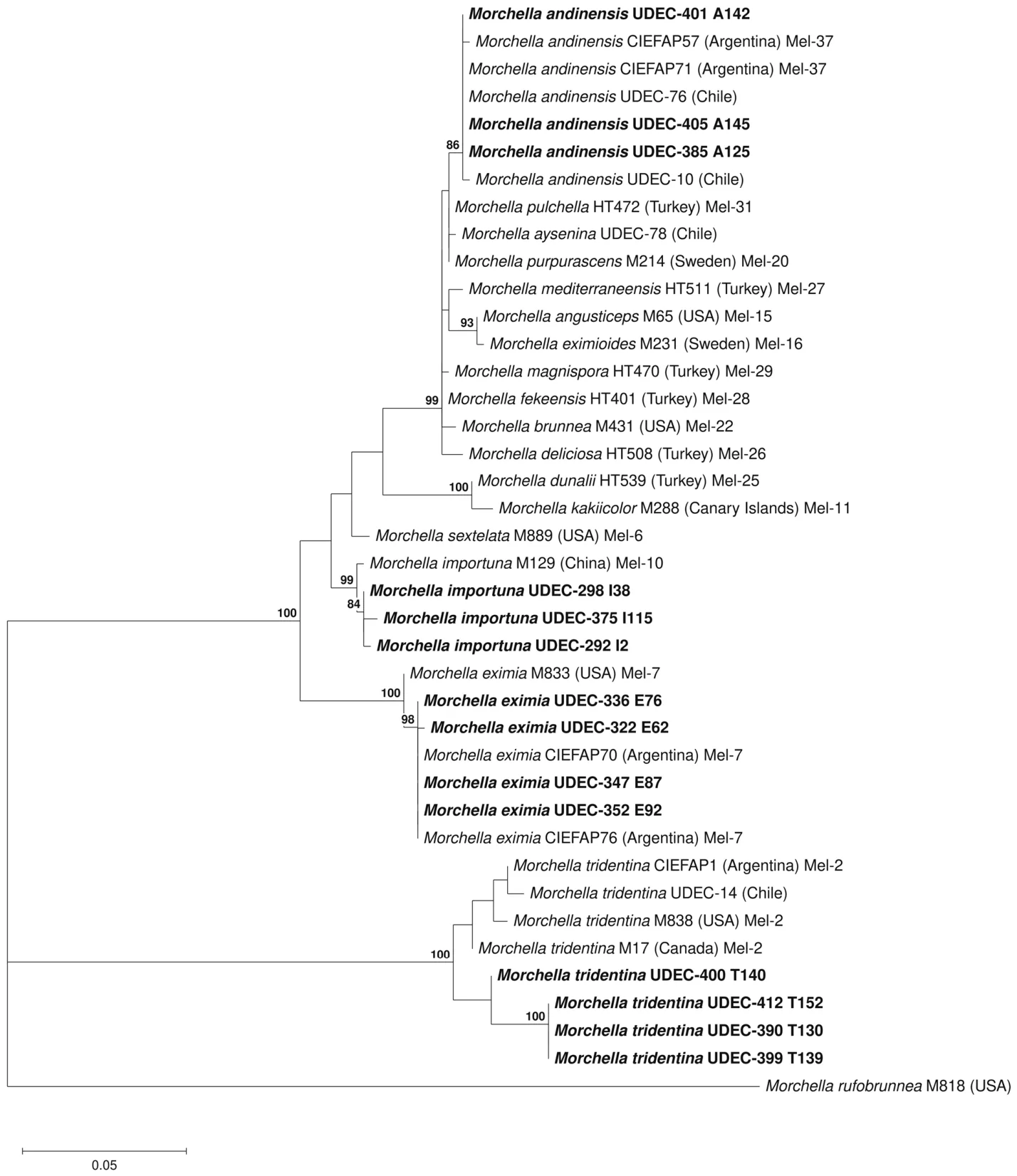
Maximum-likelihood phylogenetic tree based on ITS sequences of *Morchella* study isolates and reference sequences representing the Elata clade. Bootstrap support values based on 1000 replicates are shown at the nodes; only values ≥ 70% are displayed. Isolates included in this study are highlighted in bold and labeled with their abbreviated UDEC-LAF fungarium codes (UDEC). Species names are shown in italics. GenBank accession numbers and sequence metadata are provided in Supplementary File S2. *Morchella rufobrunnea* M818 was used as the outgroup. The scale bar represents 0.05 substitutions per site.

The morels studied here were collected from natural habitats ranging from relatively undisturbed to those exhibiting varying degrees of anthropogenic disturbance (traffic from people and domestic animals, fire, harvesting, among others). These habitats were located between 36°35′ and 37°44′ S latitude, and at elevations ranging from 95 to 806 m.a.s.l. (Table 1; Supplementary Table S1). The ITS phylogenetic reconstruction (Figure 1) placed the studied specimens within four species-level groups corresponding to *Morchella andinensis*, *M. tridentina*, *M. importuna*, and *M. eximia*, with bootstrap support of 86%, 100%, 99%, and 100%, respectively, consistent with the Elata clade framework defined by Taşkın et al. [25] and O’Donnell et al. [3]. Alignment-based identities between the studied isolates and their closest species reference sequences ranged from 99.22% to 100% (Supplementary File S2). Furthermore, the resulting species assignments were compatible with multilocus-based frameworks and previously documented distributions of Chilean morels [6,7], although the ITS reconstruction was used only to support species identification and not to infer detailed evolutionary or ecological relationships.

The species assigned to *Morchella andinensis* and *M. tridentina* were found exclusively in conserved and relatively conserved *Nothofagus*-dominated native forests (Table 1; Supplementary Table S1). This observation is consistent with the description of *M. andinensis* as a possible endemic Patagonian taxon and its previously recognized distribution in the Andean region of southern South America (47° S latitude) [6,8] and which today extends to the south-central region (37° S latitude) of the country, as evidenced by these findings. Thus, within the sites examined, both the endemic *M. andinensis* and the transcontinental *M. tridentina* were collected in native forest ecosystems at altitudes greater than 400 m (Supplementary Table S1). To date, these species have not been found in disturbed or urban environments. In the ITS phylogenetic tree, specimens of *M. andinensis* from various locations in the south-central region formed a well-defined clade with specimens from Chilean (UDEC-LAF 76 and 10) and Argentinean (CIEFAP 57 and 71) Patagonia (86% bootstrap support) and showed 100% alignment-based identity to their closest species references (Supplementary File S2). In contrast, the broader *M. tridentina* group received 100% bootstrap support, but its internal structure was weakly resolved. The subgroup containing the Argentinean, Chilean, Canadian, and USA reference sequences received 15% support, whereas the subgroup containing the south-central Chilean specimens (UDEC-LAF 400, 412, 390, and 399) received 59%. Within the latter, UDEC-LAF 412, 390, and 399 grouped with 100% support, while UDEC-LAF 400 occupied a separate internal position (Figure 1). Consequently, these internal relationships were not interpreted as geographically structured lineages.

Specimens of *M. importuna* from the south-central region grouped with the M129 reference sequence in a clade supported by 99%, while the subgroup containing the study isolates received 84% support. A similar observation was made for *M. eximia*, where the species-level group received 100% support and the subgroup comprising the Chilean study isolates and Argentinean references received 98%. Although these internal patterns were compatible with the corresponding species assignments, they were not interpreted as evidence of distinct South American lineages. Specimens of *M. importuna* and *M. eximia* were collected from disturbed habitats, such as harvested or burned forest plantations, and an urban park site (Table 1; Supplementary Table S1). This finding is consistent with previous reports from Chile [7,26] and Argentina [10], which also described *M. eximia* fruit predominantly in post-fire forests. According to the literature, *M. eximia* has been frequently characterized as pyrophilous, whereas *M. importuna* has frequently been reported in disturbed habitats, including gardens, landscaping sites, and wood-mulch beds [27,28,29,30]. *M. importuna* is currently cultivated worldwide, accounting for most of the commercial black morel production in China [31]. Its successful global dissemination has been attributed to human-mediated introduction and its high ecological plasticity, with documented establishment beyond its presumed native North American range [32]. The development of exogenous nutrient bag technology has further facilitated its large-scale cultivation [33], and physiological studies confirm its saprotrophic lifestyle under artificial conditions [18]. Taken together, the distributional patterns observed in native forests for *M. andinensis* and *M. tridentina*, in contrast to the occurrences of *M. eximia* and *M. importuna* following disturbance, align with previous reports [6,7,26,28,29,30]. Within the limits of the present sampling, these results suggest that the habitats of the endemic/native-forest taxa and transcontinental, disturbance-adapted taxa within the Elata clade, were separate, and this could be a common strategy employed by *Morchella* species found in Chile to date.

### 3.2. Initial Growth Characterization of Morchella Strains

**Figure 2:**
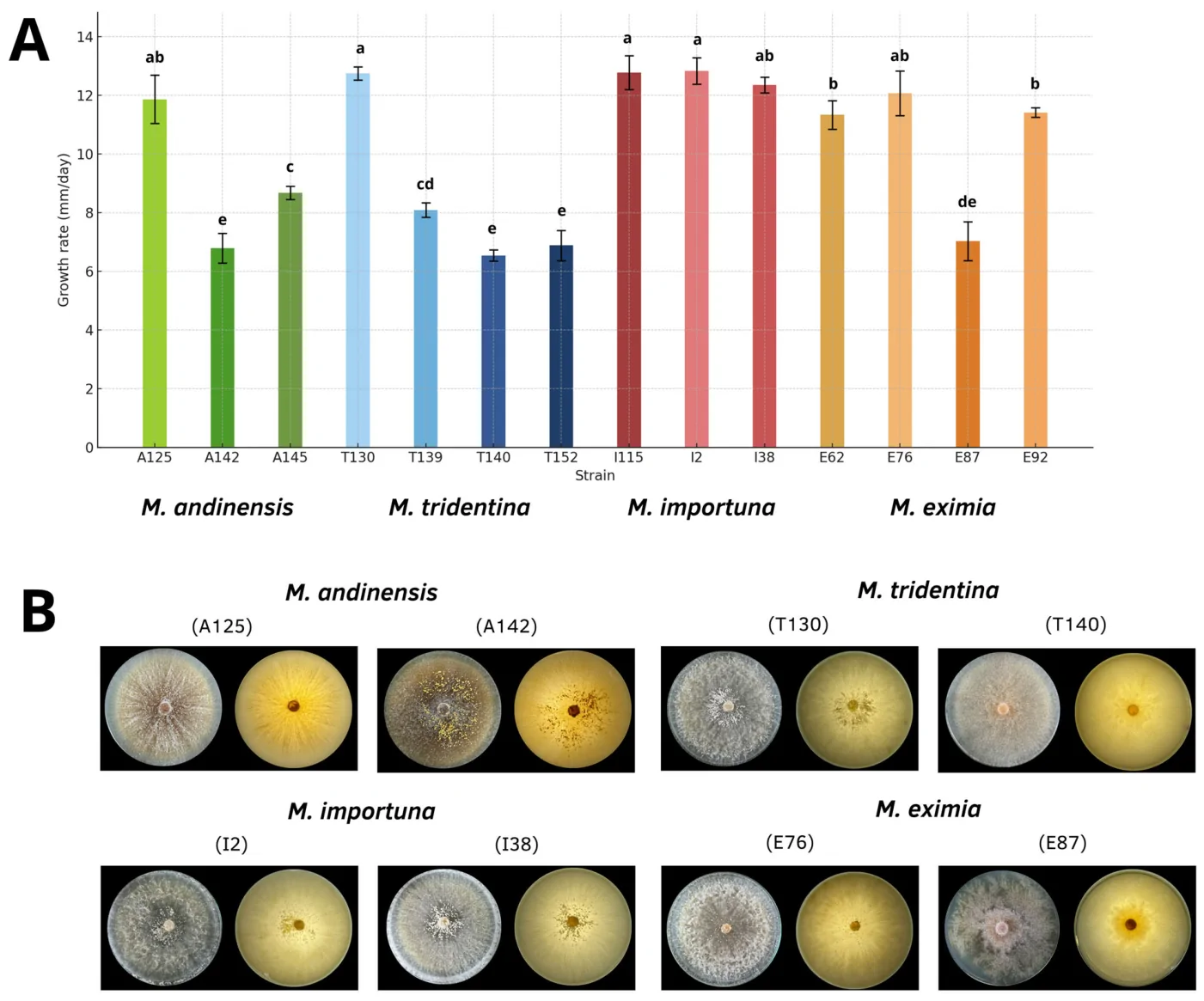
(**A**) Radial growth rate (mm·day^−1^) of *Morchella* strains cultivated on CYM (pH 7.0) at 24 °C for 10 days. Strains correspond to four species: *M. andinensis* (A125, A142, A145), *M. tridentina* (T130, T139, T140, T152), *M. importuna* (I115, I2, I38), and *M. eximia* (E62, E76, E87, E92). Bars represent mean values (*n* = 3) ± SD. Different letters above bars indicate statistically significant differences among strains according to Tukey’s HSD test. (**B**) Representative colony morphology of selected strains after 10 days of growth at 24 °C on CYM. Each strain image displays the front (**left**) and reverse (**right**) views.

Fourteen pure isolates were obtained from molecularly identified ascomata, including three isolates of *M. andinensis*, four of *M. tridentina*, three of *M. importuna*, and four of *M. eximia*. Radial growth rate on CYM at 24 °C for 10 days (Figure 2A) revealed significant differences among strains (*p* < 0.05), with rates ranging from 6.5 mm·day^−1^ (T140) to nearly 13 mm·day^−1^ (T130, I115, and I2). Considerable variation in growth rate was observed among strains from native forests, including both fast-growing (A125 and T130) and slow-growing strains (A142, T140, and T152) of *M. andinensis* and *M. tridentina*. In contrast, strains from disturbed environments (*M. importuna* and *M. eximia*), generally exhibited higher and more uniform growth rates, particularly in *M. importuna*. Strain E87 was an exception, showing the lowest performance compared to the other *M. eximia* strains. This pronounced variability is consistent with previous reports indicating significant physiological heterogeneity within the genus *Morchella* under in vitro conditions [7,15]. Comparable strain-dependent variations in radial growth rate have also been documented for *M. esculenta* and related taxa [34,35].

Based on these results, two strains of each species exhibiting contrasting growth rates were selected for subsequent assays (Figure 2B): A125 and A142 (*M. andinensis*), T130 and T140 (*M. tridentina*), I2 and I38 (*M. importuna*), and E76 and E87 (*M. eximia*). However, growth rates among *M. importuna* strains were highly similar, preventing the identification of a clearly contrasting pair; therefore, strains I2 and I38 were selected as representative isolates. The chosen strains from the different species sourced from different locations (Table 1), with the exception of *M. importuna* (I2 and I38), which were collected from a single site.

Macromorphological visual characterization revealed substantial intraspecific differences (Figure 2B). In the case of *M. andinensis*, strain A125 developed a dense, cottony aerial mycelium with a faint yellow hue, whereas A142 formed abundant, intensely pigmented sclerotia and a diffusion of dark brown pigmentation into the agar. Sclerotial development is known to reflect carbon (C) storage strategies and stress adaptability in morels [36,37]. In *Morchella* spp., sclerotia have been described as resistant resting structures that act as nutrient reservoirs, storing mainly neutral lipids that may later support fruiting body development [38]. This suggests that there may be functional differentiation between A125 and A142 strains, which were found to have significantly different growth rates. In the case of *M. tridentina*, only strain T130 produced central sclerotia, whereas T140, which exhibited a reduced growth rate, lacked visible reserve structures. The two *M. importuna* strains (I2 and I38) showed similar morphology, characterized by moderately cottony white mycelium and central white sclerotia, consistent with descriptions of vigorous saprotrophic growth and C acquisition in cultivated lineages of this species [11,33]. However, sclerotial formation should not be interpreted based solely on rapid radial growth. Studies in *M. esculenta* demonstrated that sclerotial differentiation also depends on nutrient location, sugar type and concentration, and physiological nutrient translocation between the mycelium and developing sclerotia [23,39]. The greatest morphological variability was observed in the cultures of both strains of *M. eximia*. While strain E76 formed uniform, cottony mycelium with limited sclerotia, E87 grew irregularly and failed to produce sclerotia, in line with its lower growth rate. This behavior is similar to that observed in strain T140 of *M. tridentina*, where the absence of sclerotia was accompanied by low mycelial growth rate. Together, quantitative growth rate parameters and qualitative colony traits indicate that *Morchella* strains exhibit marked differences.

### 3.3. Performance of Morchella Strains on Different Carbon (C) Supplements

**Figure 3:**
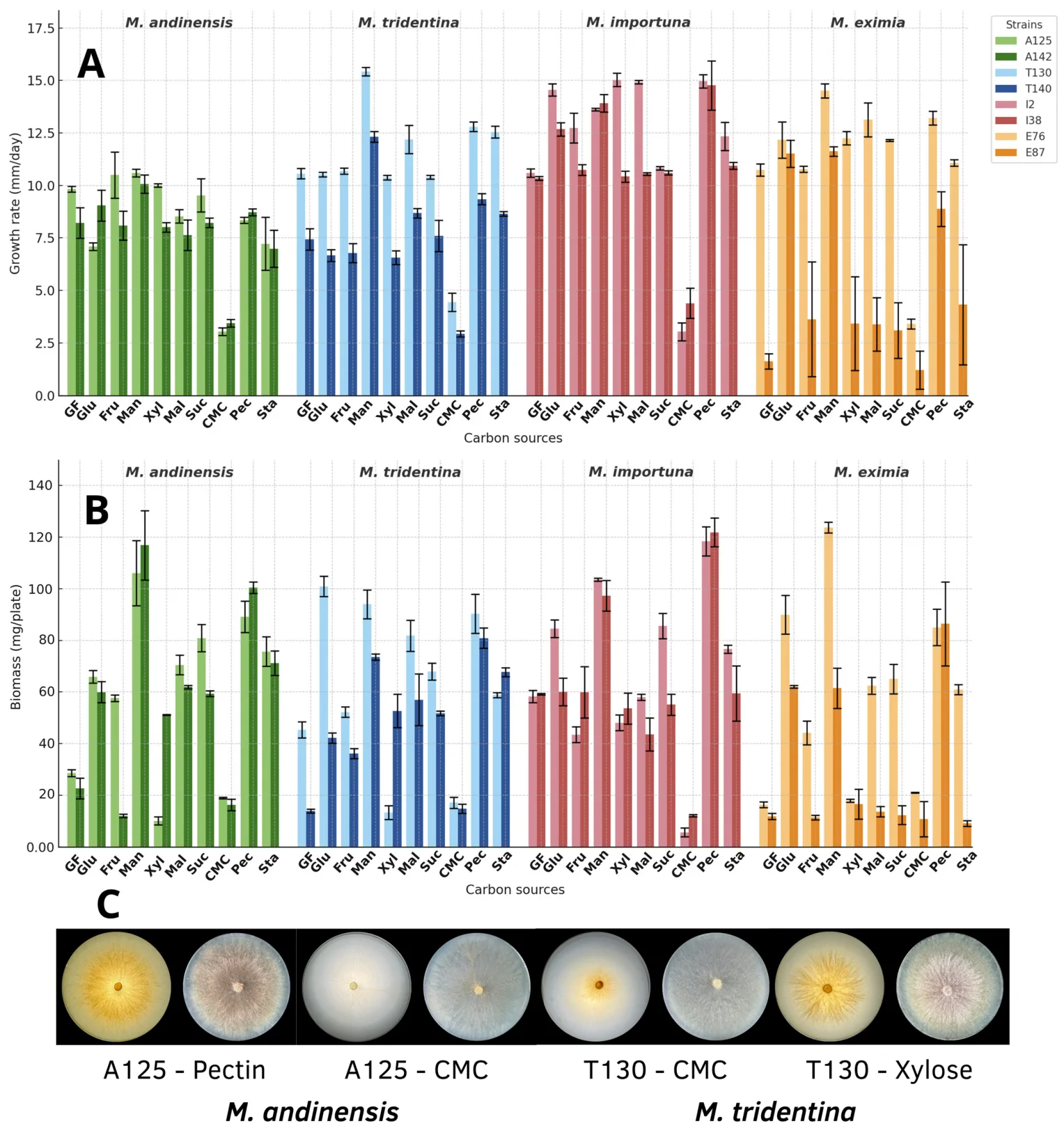
Effects of carbon supplements on development of *Morchella* strains after 10 days at 24 °C. (**A**) Radial growth rate (mm·day^−1^) and (**B**) biomass accumulation (mg plate^−1^) on each carbon source. Bars are mean (*n* = 3) ± SD. (**C**) Representative colony development of A125 and T130 strains on selected carbon sources (front and reverse views). Carbon sources: Glucose-free medium (GF), glucose (Glu), fructose (Fru), mannose (Man), xylose (Xyl), maltose (Mal), sucrose (Suc), carboxymethylcellulose (CMC), pectin (Pec), and starch (Sta).

The in vitro performance of the previously selected *Morchella* strains (Section 3.2) was evaluated using CYM supplemented with a range of C supplements, including monosaccharides (glucose, mannose, fructose, and xylose), disaccharides (maltose and sucrose), and polysaccharides (carboxymethylcellulose, pectin, and starch). Two-way ANOVA revealed highly significant effects of strain, carbon source, and their interaction on both radial growth rate and biomass accumulation (all *p* < 0.0001; Supplementary File S1). This interaction was also evident in the strain-level responses, since differences among strains were maintained across several C sources but became less pronounced under more complex substrates, such as carboxymethylcellulose (CMC), where most strains exhibited the lowest growth rates (Figure 3A,B). Notably, all strains grew in the absence of a readily metabolized C source (glucose-free medium), although with varying degrees of success. This suggests that, in the absence of glucose, the *Morchella* strains may use peptone and/or yeast extract from the CYM as alternative C sources. Growth rates ranged from 1.2 ± 0.91 mm·day^−1^ in CMC medium (*M. eximia* E87) up to 15.4 ± 0.2 mm·day^−1^ on mannose (*M. tridentina* T130) (Figure 3A). Mannose supported the fastest average growth (12.8 mm·day^−1^), followed by pectin (11.4 mm·day^−1^), while glucose and maltose formed a second tier around 10.0 mm·day^−1^ (Supplementary File S1). CMC consistently yielded the lowest growth rates in all strains (Figure 3A). Although E87 was the least vigorous strain overall, its reduction was not uniform across all C sources, since comparatively better growth was observed on glucose, mannose, and pectin. This trend is consistent with previous reports indicating the efficient growth of *Morchella* spp. on hexoses, such as glucose and mannose [21,34]. Among the polysaccharides used as supplements, pectin and starch stimulated greater growth responses in *Morchella* strains than CMC. The consistently poor performance of all strains on a CMC-supplemented medium suggests that a reduced growth response to this polysaccharide is likely a shared trait among the *Morchella* strains evaluated. According to previous reports on *M. importuna*, the cellulose utilization appears to be limited [18]. Similarly, during the decomposition of exogenous nutrient bags by *M. importuna*, amylose and amylopectin were extensively metabolized, whereas cellulose and hemicellulose showed comparatively lower consumption [33], and this is further supported by Reddy and Kanwal [40], who noted relatively low cellulase activity in *M. spongiola* grown on different lignocellulosic substrates.

The maximum biomass accumulation values (up to 120 mg·plate^−1^) were recorded for strain E76 of *M. eximia* on mannose and both strains of *M. importuna* on pectin over a 10-day incubation period (Figure 3B). Minimal biomass accumulation (<30 mg·plate^−1^) was detected in several strains on CMC, xylose, or glucose-free medium, particularly in strain E87 from *M. eximia*. In general, biomass showed a more variable response than radial growth rate, especially in *M. eximia* and, to a lesser degree, in *M. andinensis*. Some C supplements produced relatively high colony expansion while yielding a proportionally lower biomass, indicating that rapid radial growth did not always correlate with dense mycelial production. This trend was evident, for example, in some responses to maltose, sucrose, or starch, whereas mannose and pectin more frequently promoted both radial expansion and biomass accumulation. Similar substrate-dependent effects on growth and differentiation have been documented in other *Morchella* species [18,41].

The boxplots revealed clear strain-level differences. *M. importuna* strains (I2 and I38) showed the highest medians and relatively narrow interquartile ranges (IQR) across C sources, indicating comparatively low dispersion among the observed responses (Figure S1A,B). In contrast, strain E87 from *M. eximia* exhibited consistently low medians and greater dispersion, reflecting reduced growth capacity across C supplements. *M. andinensis* and *M. tridentina* displayed intermediate behavior, with pronounced increases on mannose and pectin but lower growth on CMC and glucose-free medium. Previous research has documented intra- and interspecific variability in *Morchella* isolates cultivated on different C sources [21,42], underscoring the need for strain-level assessment.

Carbon supplements produced only moderate changes in colony morphology, with overall colony architecture remaining relatively conserved (Figure 3C). Mannose and pectin generally promoted uniform radial expansion with well-developed aerial mycelium. In contrast, CMC resulted in thinner, less dense colonies with reduced aerial growth, as observed in *M. andinensis* A125 and *M. tridentina* T130 (Figure 3C). In many cases, xylose and glucose-free treatments often produced colonies that were more compact and pigmented. After 10 days, no sclerotia were observed in any C supplements across strains. These morphological differences were consistent with the quantitative trends observed for growth rate and biomass accumulation and agree with previous observations that C composition might affect mycelial differentiation and development in *Morchella* [34,41]. Overall, the results suggest that the effect of C supplements on these morel strains is dependent on the substrate and varies among genotypes.

### 3.4. Performance of Morchella Strains on Different Nitrogen (N) Supplements

The in vitro behavior of the different *Morchella* strains was tested using CYM supplemented with various N sources. These included simple organic (alanine and proline) or complex (peptone) sources, and inorganic sources (ammonium nitrate and ammonium sulfate). The type of N source was found to be another key factor in *Morchella* growth, exhibiting a strain-dependent response similar to the previously noted effect of the C source type. A two-way ANOVA revealed highly significant effects of N sources, strain, as well as strain × source interactions, on both growth rate and biomass accumulation (all *p* < 0.0001; Supplementary File S1). Organic N supplements typically promoted faster growth and/or more biomass accumulation than the inorganic sources. Peptone yielded the highest mean growth rate (10.53 mm·day^−1^), closely followed by amino acids, whereas ammonium sulfate resulted in the slowest mean growth rate (7.63 mm·day^−1^) (Supplementary File S1).

**Figure 4:**
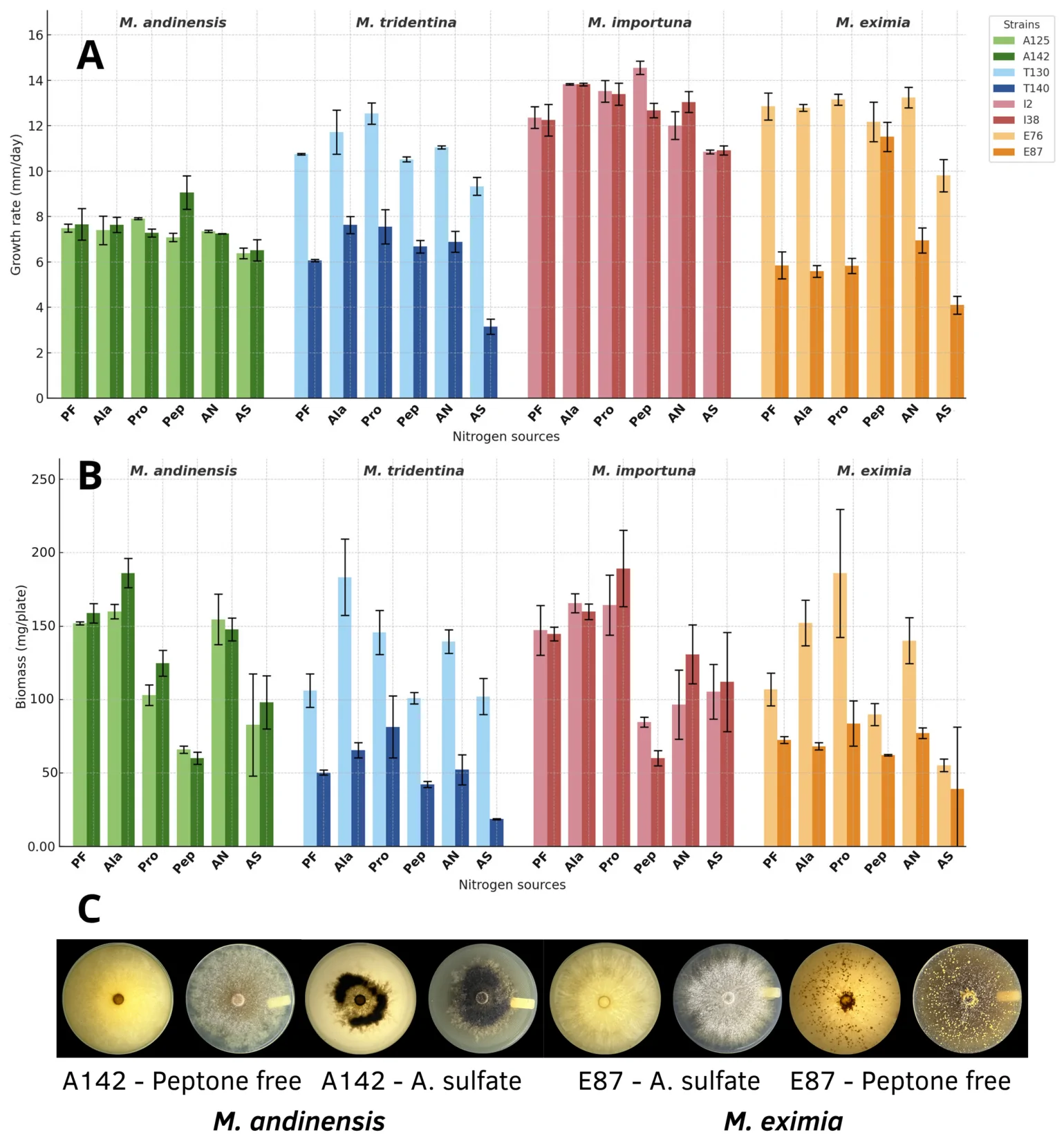
Effects of nitrogen supplements on development of *Morchella* strains after 10 days at 24 °C. (**A**) Radial growth rate (mm·day^−1^) and (**B**) biomass accumulation (mg plate^−1^) on each nitrogen source. Bars are mean (*n* = 3) ± SD. (**C**) Representative photographs of colony development of A142 and E87 strains on selected nitrogen conditions (front and reverse views). Nitrogen sources: peptone-free medium (PF), alanine (Ala), proline (Pro), peptone (Pep), ammonium nitrate (AN), and ammonium sulfate (AS).

The boxplots demonstrated that both strains of *M. importuna* (I2, I38) and the E76 strain of *M. eximia* showed the fastest overall growth across the N treatments (average ≥ 12.3 mm·day^−1^); in contrast, *M. tridentina* T140 and *M. eximia* E87 exhibited consistently slower growth rates (<7 mm·day^−1^) (Figure 4A; Supplementary File S1). Boxplots illustrate that strains I2 and I38 achieved high median growth with very narrow IQRs across N sources, suggesting efficient growth on diverse N forms. Conversely, T140 and E87 strains displayed lower medians and broader variability (Figure S1A,B).

Despite the rapid mycelial growth observed with peptone (Figure 4A), the accumulation of biomass was negatively affected by this N supplement across strains (Figure 4B). In contrast, most of the strains exhibited the highest mycelial biomass when supplied with amino acids. Peptone, a complex organic N source composed mainly of peptides and amino acids, may promote rapid exploratory growth. However, further enzymatic processing may be required prior to the efficient incorporation of N into cellular biomass [18,33]. Secretomic evidence showed that peptone utilization in *M. importuna* involves marked changes in extracellular enzyme profiles, including proteases and peptidases [18]. On the other hand, Prasher et al. [42] reported that L-glutamic acid supported the highest mycelial dry weight in *M. hybrida* among the organic N sources evaluated. The lowest biomass accumulation was observed with ammonium sulfate, particularly in strains *M. tridentina* T140 and *M. eximia* E87, which demonstrated consistency in terms of low biomass and growth rate, regardless of the N supplements (Figure 4A,B). Importantly, strain E87 also demonstrated the most deficient physiological responses in prior experiments with different C sources (Figure 3A,B). Although there was a significant difference in radial growth rate between the *M. andinensis* and *M. importuna* (Figure S1B,C; Supplementary File S1), this difference was not reflected in biomass accumulation (Figure 4A,B). In addition, no significant differences were observed between the two strains of these species (Figure S1C, Supplementary File S1). In contrast, marked intraspecific differences were observed in *M. tridentina* and *M. eximia* with respect to radial growth rate and biomass production. The observed growth in the peptone-free medium suggested that the N and growth factors supplied by the yeast extract in the CYM were probably sufficient to sustain short-term mycelial growth in most *Morchella* strains (Figure 4A,B). Reddy and Kanwal [40] evaluated the growth of *M. spongiola* in mineral salts broth, finding that yeast extract significantly improved biomass production relative to the other sources studied when tested as a replacement N source.

More substantial alterations in mycelial morphology were observed under N supplements than under C supplements, among different strains. In some strains, such as *M. eximia* E87, reduced radial growth was accompanied by intensified browning of the colony and/or medium, particularly under ammonium sulfate conditions (Figure 4C). Conversely, *M. andinensis* A142 exhibited relatively uniform growth in the presence of ammonium sulfate, whereas the peptone-free medium supported the formation of numerous small brown compact structures, indicative of early sclerotial differentiation (Figure 4C). Similar structures were also observed in peptone-free media in strains A125, I2, and I38. These results suggest that the presence of different N supplements in the culture medium promoted localized hyphal aggregation and sclerotium structure formation in some *Morchella* strains. This differs from the observations made with C sources, where no sclerotium formation occurred, at least during the 10-day cultivation period. These results align with research on *M. hybrida* nutrition, which demonstrated that sclerotial morphogenesis is selectively triggered by specific N sources, including both ammonium salts and select amino acids [42].

### 3.5. Performance of Morchella Strains Under Different pH Conditions

**Figure 5:**
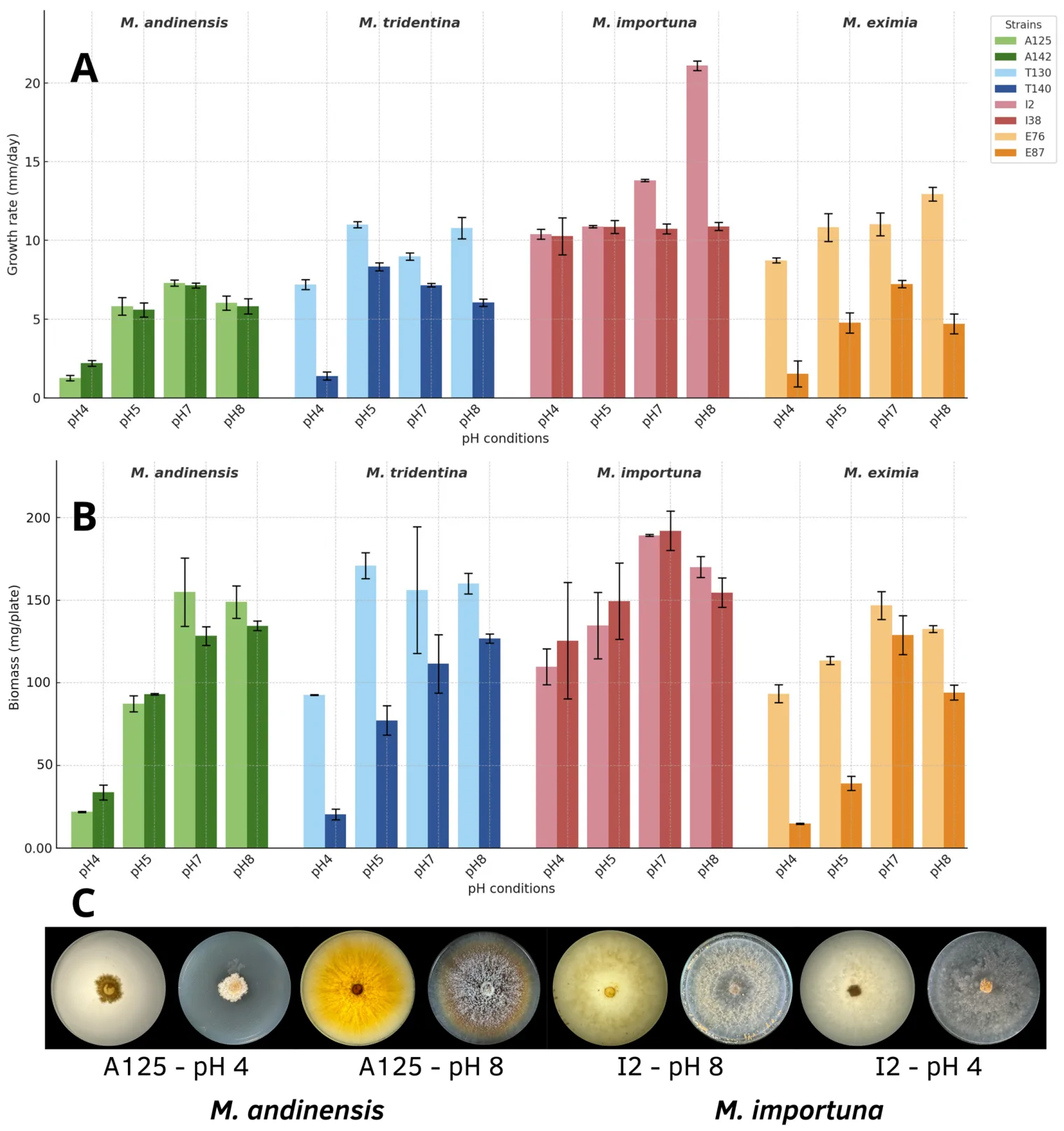
Effects of pH on development of *Morchella* strains after 10 days at 24 °C. (**A**) Radial growth rate (mm·day^−1^) and (**B**) biomass accumulation (mg plate^−1^) at each pH condition. Bars are mean (*n* = 3) ± SD. (**C**) Representative photographs of colony development of I2 and A125 strains under selected pH conditions (front and reverse views). Treatments are identified by their target pH values (pH 4, 5, 7, and 8); the corresponding values measured on the agar surface after autoclaving were 4.5, 5.5, 7.2, and 8.5, respectively. Accordingly, axis labels denote the target rather than the measured pH values.

As with the different C and N supplements, the initial pH value of the CYM had a significant influence on the in vitro performance of *Morchella* strains, with strain-dependent responses in both radial growth rate and biomass accumulation. Two-way ANOVA revealed highly significant effects of strain, pH, and their interaction on both variables (all *p* < 0.0001; Supplementary File S1). The most favorable pH conditions for most of the strains were neutral (pH 7.2) to alkaline (pH 8.5), whereas acidic conditions (pH 4.5) significantly reduced radial growth rate in some strains, particularly A125 and A142 from *M. andinensis*, T140 from *M. tridentina*, and E87 from *M. eximia* (Figure 5A; Supplementary File S1). Mean radial growth rate was highest at pH 8.5 (9.79 mm·day^−1^), followed by pH 7.2 and pH 5.5 (9.18 and 8.51 mm·day^−1^, respectively), while growth dropped markedly at pH 4.5 (5.37 mm·day^−1^) (Supplementary File S1). The boxplots demonstrate a strain-level trend in the radial growth rate across pH treatments, where I2 showed the highest median and upper range, followed by I38 and E76. The strains A125, A142, T140, and E87 were among the lowest median growth rates (Figure S1E). The growth rate exhibited a comparable trend with biomass accumulation (Figure 5A,B), with maximal values recorded between pH 7.2 and 8.5, particularly in *M. importuna* strains. Conversely, pH 4.5 was associated with growth reduction. The biomass boxplots reinforced this separation, with strains I2, I38, and T130 among the highest biomass producers, whereas strains T140 and E87 remained among the lowest producers across different pH values (Figure S1F). In this assay, *M. importuna* was the only species that demonstrated consistent development across the entire pH range evaluated, with the I2 strain exhibiting the highest radial growth rate (21.1 mm·day^−1^) at pH 8.5 (Figure 5C).

These findings are consistent with previous studies reporting optimal development of certain *Morchella* species under neutral to slightly alkaline conditions [21,34]. Kalyoncu et al. [43] evaluated six *Morchella* species and reported optimal mycelial growth between pH 6 and 7, with reduced growth above pH 7.5 and strong inhibition under acidic conditions (pH ≤ 5.5). Similarly, Prasher et al. [42] reported maximal mycelial growth and sclerotia production of *M. hybrida* at pH 7.0, with inhibition of sclerotial formation under acidic conditions (pH ≤ 4). The consistent response of *M. importuna* in vitro under alkaline conditions is also compatible with field observations indicating favorable performance of cultivated morels in slightly alkaline soils [44]. The initial pH of CYM produced changes in colony morphology, with responses varying among strains and species (Figure 5C). In certain strains, a marked pigmentation of the mycelium and the culture medium was noted, depending on the pH level. In other cases, at pH 8.5, the development of sclerotia was observed, specifically in strains A125 and I2 (Figure 5C).

### 3.6. Performance of Morchella Strains at Different Incubation Temperatures

**Figure 6:**
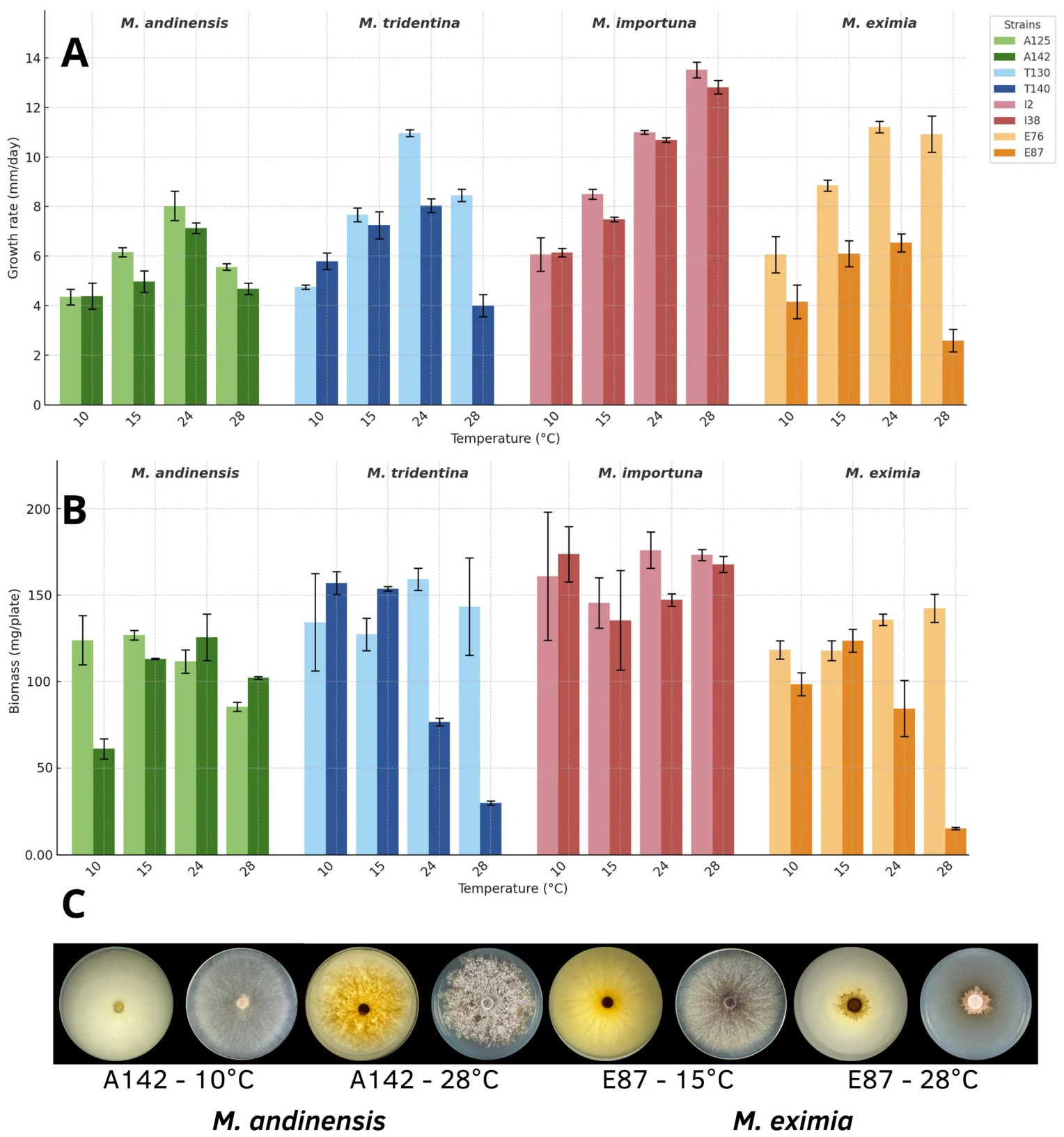
Effects of temperature on development of *Morchella* strains after 10 days. (**A**) Radial growth rate (mm·day^−1^) and (**B**) biomass accumulation (mg plate^−1^) at each temperature. Bars are mean (*n* = 3) ± SD. (**C**) Representative photographs of colony development of A142 and E87 strains under selected temperature conditions (front and reverse views).

Temperature significantly affected *Morchella* development, with strain-dependent responses in both radial growth rate and biomass accumulation. Two-way ANOVA revealed highly significant effects of strain, temperature, and their interaction on both variables (all *p* < 0.0001; Supplementary File S1). Considering the mean response of all strains, 24 °C resulted in the highest radial growth, with a mean of 9.2 mm·day^−1^ (Supplementary File S1). However, this general trend was not uniform across species. Both *M. importuna* strains demonstrated increased growth at temperatures of up to 28 °C, whereas the E76 strain from *M. eximia* exhibited sustained high growth at 24 and 28 °C (Figure 6A). In contrast, *M. andinensis*, *M. tridentina*, and the E87 strain from *M. eximia* exhibited heightened sensitivity at 28 °C. Additionally, at low temperatures (10 and 15 °C) the growth rate across the strains was also reduced (Figure 6A). The boxplots reinforced these strain-level differences. Both *M. importuna* strains showed the highest median growth rate and broad upper ranges. In contrast, the E87 strain from *M. eximia* exhibited the lowest growth rate and showed marked sensitivity to high temperature (Figure S1G). These results are consistent with previous reports indicating that *Morchella* spp. often grow optimally around 20 to 24 °C under in vitro conditions in solid medium [7,21,36,44]. The high growth rates of strains I2 and I38 at 28 °C suggest that certain *M. importuna* genotypes can maintain, or even increase, growth at temperatures that adversely affect other species. At 10 °C, radial growth rate was reduced in all strains by around 40% compared to growth at 24 °C. However, this temperature condition did not completely inhibit growth. This is relevant because soil temperatures during field development of *M. importuna* have been reported within relatively low ranges, around 6 to 15 °C [33,45].

No significant differences in biomass production were noted for most strains at temperatures of 10, 15, and 24 °C (Supplementary File S1). At 28 °C, a significant reduction in biomass production was only observed in the most sensitive T140 and E87 strains (Figure 6B; Supplementary File S1). The biomass boxplots showed that I2 and I38 were among the highest biomass producers, whereas the E87 strain was clearly separated among the lowest values (Figure S1H). Therefore, a high radial growth rate did not always correspond directly to maximum biomass accumulation, suggesting that temperature affected colony expansion and mycelial biomass formation in partially different ways.

Representative colony images showed that temperature affected not only radial expansion but also colony morphology and pigmentation (Figure 6C). Overall, colonies subjected to restrictive thermal conditions exhibited altered development, characterized by less peripheral expansion, stronger yellow to brown pigmentation, and less uniform mycelial growth. For instance, *M. andinensis* A142 showed marked yellow-brown pigmentation and abundant compact mycelial aggregates with superficial cottony development structures at 28 °C, indicating a clear temperature-induced shift in morphology. In contrast, the growth of *M. eximia* E87 was strongly restricted at 28 °C, forming a compact and irregular colony with intense pigmentation and limited expansion. These visual patterns support the quantitative evidence that temperature responses were strongly strain-dependent (Figure 6C).

### 3.7. Integrated Strain Performance and Principal Component Analysis (PCA)

**Figure 7:**
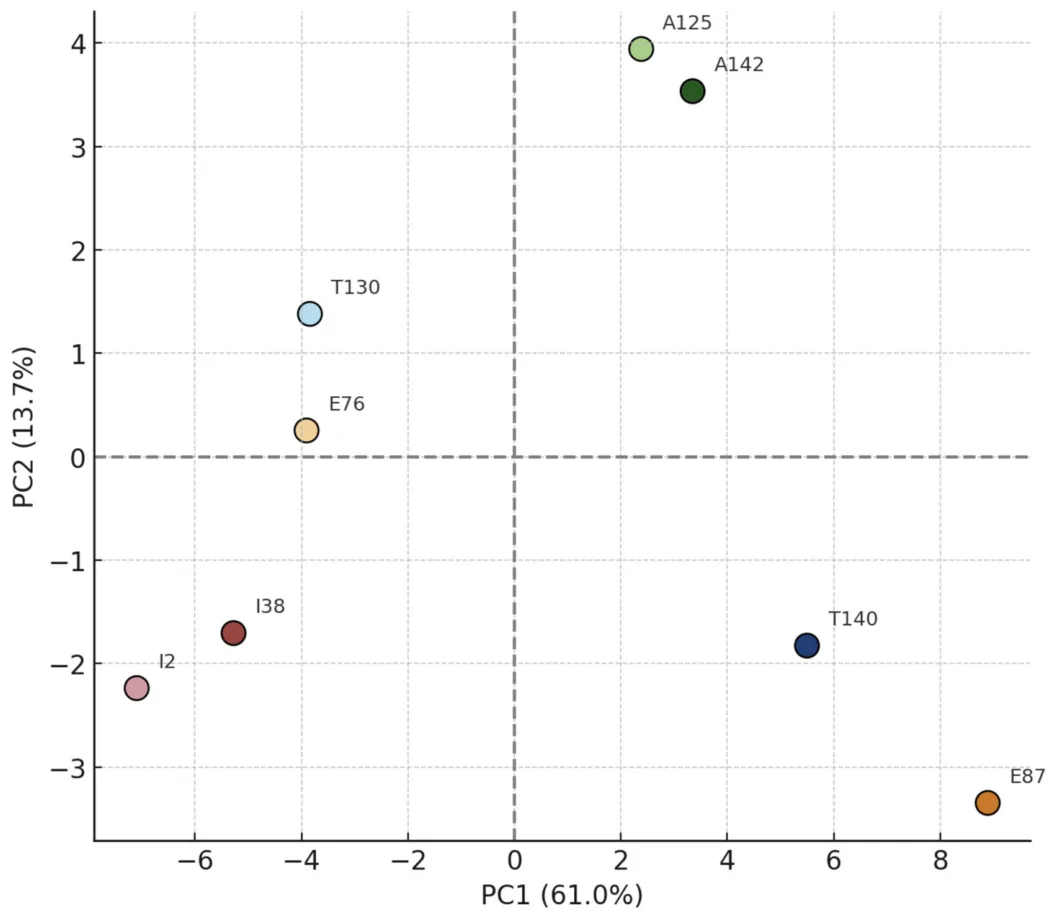
Principal component analysis (PCA) of *Morchella* strains under different in vitro conditions. The analysis was performed on a strain × variable matrix comprising eight strain-level observations and standardized mean values of radial growth rate (mm·day^−1^) and biomass accumulation (mg·plate^−1^), calculated from three replicates per condition. The treatments included target pH (4, 5, 7, and 8), temperature (10, 15, 24, and 28 °C), carbon sources (GF, Glu, Fru, Man, Xyl, Mal, Suc, CMC, Pec, and Sta), and nitrogen sources (PF, Ala, Pro, Pep, AN, and AS). PCA scores: strains are shown as colored circles according to species and strain: *M. andinensis*, A125 (light green) and A142 (dark green); *M. tridentina*, T130 (light blue) and T140 (dark blue); *M. importuna*, I2 (light red) and I38 (dark red); and *M. eximia*, E76 (light brown) and E87 (orange). Dashed lines indicate zero values on PC1 and PC2. The PCA was interpreted as an exploratory representation of similarities and differences among standardized strain response profiles.

An exploratory principal component analysis (PCA) performed on standardized mean radial growth rate and biomass accumulation values across all treatments showed that the first two components explained 74.7% of the total variance (PC1 = 61.0%, PC2 = 13.7%) (Figure 7). The strains of *M. importuna* (I2 and I38) were found to be distributed along the negative extreme of PC1, whereas the E87 strain of *M. eximia* was clearly positioned on the opposite side (Figure 7). The remaining strains occupied intermediate positions, with *M. tridentina* T140 strain displaced toward the side associated with comparatively lower responses than its conspecific T130 strain. Conversely, strains A125 and A142 from *M. andinensis* were grouped separately along PC2.

The loading distribution revealed that several variables linked to an increased radial growth rate and biomass accumulation were oriented toward the negative side of PC1, in accordance with the positioning of the *M. importuna* strains (Figure S2). Variables contributing to this general pattern included radial growth responses under several N and temperature conditions, together with biomass responses across C, N, pH, and temperature treatments. No individual variable alone explained the distribution along PC1, indicating that this component reflected a broad pattern of covariation among multiple in vitro responses. The positive side of PC1 was associated with comparatively lower responses across several conditions, particularly the E87 strain and, to a lesser extent, the T140 strain. Thus, PC1 mainly summarized overall gradient in the standardized physiological responses across the in vitro conditions evaluated, rather than a specific response to an individual treatment. The separation of strains A125 and A142 from the remaining strains along PC2 suggests a distinct response pattern across the evaluated conditions. PC2 was more strongly associated with biomass-related variables under several C and N conditions, in contrast to its association with radial growth and biomass responses under low-temperature conditions. Despite not consistently exhibiting the fastest radial growth rates, these strains often showed comparatively high biomass accumulation across several treatments. Their position in the multivariate space may therefore reflect a different combination of radial growth and biomass responses compared with the other strains, rather than differences in overall performance alone. However, given that both growth rate and biomass variables contributed to this axis, this pattern should be interpreted as an exploratory indication of differential response profiles rather than as evidence that strains clearly prioritize either colony expansion or biomass production.

The exploratory PCA provided a multivariate summary consistent with the strain-level differences detected in the individual assays. Strains I2 and I38 occupied nearby positions in the PCA space and originated from the same harvested *P. radiata* plantation (Table 1). However, the analysis cannot determine whether this similarity reflects their shared origin, the selection procedure, or individual physiology (Table 1). This observation also cannot be generalized, as other evaluated strains did not show a consistent relationship between origin and physiological similarities. For example, the two *M. andinensis* strains were also grouped relatively close to each other, despite being obtained from ascomata collected from different native forest locations, which are geographically distant from each other (Supplementary Table S1). These results underscore the importance of evaluating multiple strains per species and, ideally, strains from geographically distinct environments, since species identity, origin, and strain-specific physiology may all contribute to variation in in vitro culture responses.

### 3.8. Sclerotia Formation Under Split-Plate Conditions

Sclerotia formation was evaluated because these structures have been related to developmental and nutrient storage in *Morchella*, with implications for survival in extreme conditions, reserve accumulation, and fruiting body formation under controlled conditions [14]. They have been described as nutrient reservoirs that support subsequent reproductive development, making their induction a potentially useful criterion for physiological characterization and strain selection for cultivation purposes [7,23,38]. In this study, a split-plate method [23] was used to generate nutritional asymmetry between a side with CYM deficient in nutrients and a standard CYM side. This approach enabled the evaluation of sclerotia formation by different strains under localized nutrient imbalance. Considerable variation in sclerotia formation was observed among replicate plates in this assay. For this reason, the amounts are expressed as a range rather than a mean (Table 2; Supplementary Table S2).

**Figure 8:**
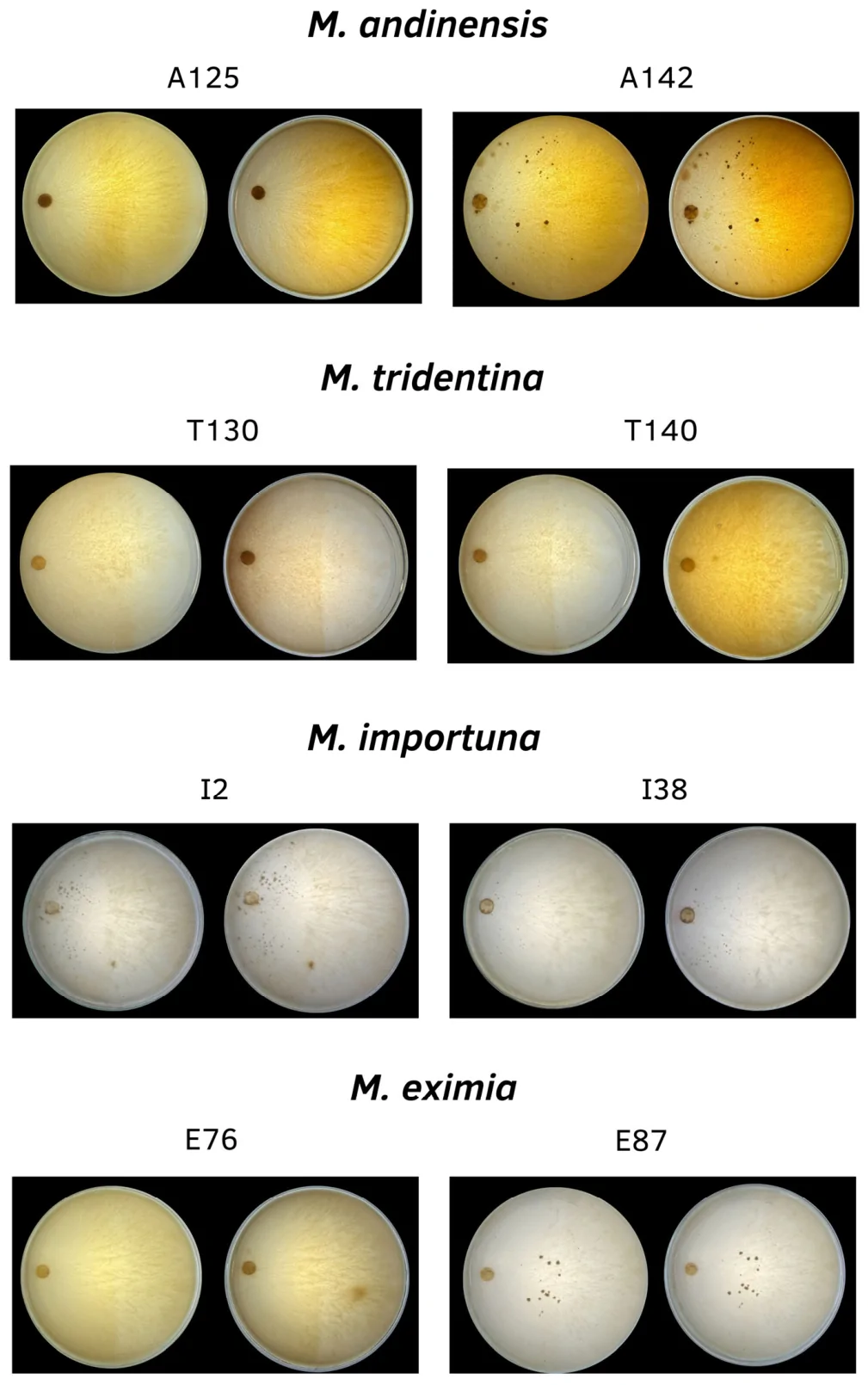
Colony morphology and sclerotia development of *Morchella* strains grown under split-plate conditions at 24 °C after 14 days (**left**) and 21 days (**right**) of incubation. Each plate (90 mm) contained a nutrient-deficient CYM side and a standard CYM side. The agar plug (8 mm) used as inoculum was placed on the nutrient-deficient side.

After 14 days of incubation under split-plate conditions, clear differences were observed in sclerotia formation by *Morchella* strains (Table 2). *M. andinensis* A125, *M. tridentina* T130 and T140, and *M. eximia* E76 did not produce sclerotia under these induction conditions, whereas *M. andinensis* A142 and *M. eximia* E87 yielded low to moderate amounts of sclerotia on the nutrient-deficient side. Structures produced by A142 were predominantly large (>1.5 mm), yellow, and present in both superficial and submerged positions on the solid medium, whereas those produced by E87 were predominantly large, orange, and superficial. In contrast, both strains of *M. importuna* showed notably higher production capacity, with I2 producing the highest quantity of sclerotia, followed by I38. In these strains, sclerotia were small to medium (≤1.5 mm), white, and mostly submerged in the agar (Table 2). Colony development was monitored until 21 days (Figure 8). During this period, no substantial increase in the number of sclerotia was observed compared with day 14. The principal changes were a slight increase in apparent size and progressive intensification of pigmentation, particularly in strains A142 and E87. This suggests that sclerotia induction occurred early and was followed mainly by maturation and pigmentation rather than numerical increase.

The predominance of sclerotia under nutrient-deficient conditions suggests that localized nutrient limitation or resource imbalance can promote the differentiation of compact mycelial structures [39]. However, the response was clearly strain-dependent. The abundant production observed in strains I2 and I38 from *M. importuna* indicates a high capacity for sclerotia formation from the mycelium. In contrast, the complete absence of sclerotia in the A125, T130, T140, and E76 strains indicates that this response was not generalized across the evaluated strains, at least until 21 days of evaluation. This result is particularly relevant because A125, T130 and E76 strains showed sclerotial development under previous growth conditions (Section 3.2, Section 3.3 and Section 3.4), suggesting that sclerotia formation in these strains depends strongly on the culture conditions and not only on intrinsic genotype. In addition, the previous observation of abundant sclerotia in the A142 strain cultivated in peptone-free medium and in the temperature assays (Section 3.4 and Section 3.6) is consistent with its positive response under the split-plate assay. The absence of sclerotia in strains that had previously produced these structures may be related to the specific growth conditions required by certain strains. In this induction assay, the strains were inoculated onto a depleted medium, which may have affected their growth and, consequently, their ability to form sclerotia during the evaluation period. Overall, this assay indicated that sclerotia production under nutrient-partitioned conditions is highly strain-dependent, with *M. importuna* strains exhibiting the greatest formation capacity, while other strains displayed reduced, absent, or condition-dependent formation.

## 4. Conclusions

This study provides an integrated in vitro characterization of the physiological responses of Chilean *Morchella* strains isolated from native forests, disturbed forest plantations, and urban disturbed environments. Overall, the results indicate that the mycelial performance of the selected strains of the endemic *M. andinensis* and the transcontinental *M. tridentina*, *M. importuna* and *M. eximia* varied among strains, and therefore, cannot be reliably inferred from species identity alone. The different responses observed under various in vitro culture conditions highlight the importance of strain-level evaluation when assessing growth rates, biomass production, and sclerotial development, for the purpose of screening strains for future studies on cultivation or preservation.

The combined in vitro evaluation of C and N supplements, pH, temperature, and multivariate performance revealed distinct physiological responses among the selected strains. The two *M. importuna* strains generally showed comparatively high radial growth rates and biomass responses, and sclerotia formation capacity. In contrast, *M. eximia* E87 and *M. tridentina* T140 exhibited the least favorable performance across all assays. Meanwhile, the strains from endemic *M. andinensis* exhibited intermediate behavior. The exploratory PCA provided a descriptive summary of the variation among the standardized response profiles of the eight selected *Morchella* strains. Most of the evaluated strains differed in terms of origin. However, the two *M. importuna* strains shared a similar origin, which may partly explain the limited differences observed between strains within this species and the greater variability detected in the remaining strains.

These results provide valuable information to guide the improvement of preservation methods in the Chilean *Morchella* culture collection of strains, particularly the endemic species *M. andinensis*. Furthermore, the mycelial characterization of *Morchella* strains can support the development of cultures for diverse biotechnological purposes.

## Figures and Tables

**Table 1:** Specimens used in this study and collection sites.

Specimen Code	Strain Code	Species Identified	GenBank Accession	Collection Date	Condition/Location
**UDEC-LAF 385**	**A125**	*M. andinensis*	PZ448500	October 2022	Conserved relic of the native *Nothofagus* forest/Villucura, Biobío Region
**UDEC-LAF 401**	**A142**	*M. andinensis*	PZ448498	October 2022	Native *Nothofagus* forest; Pemuco, Ñuble Region
UDEC-LAF 405	A145	*M. andinensis*	PZ448499	October 2022	Native *Nothofagus* forest; Pemuco, Ñuble Region
**UDEC-LAF 390**	**T130**	*M. tridentina*	PZ448495	October 2022	Native *Nothofagus* forest; Peralillo, Biobío Region
UDEC-LAF 399	T139	*M. tridentina*	PZ448497	October 2022	Native *Nothofagus* forest; Pemuco, Ñuble Region
**UDEC-LAF 400**	**T140**	*M. tridentina*	PZ448496	October 2022	Native *Nothofagus* forest; Pemuco, Ñuble Region
UDEC-LAF 412	T152	*M. tridentina*	PZ448494	October 2022	Native *Nothofagus* forest; Pemuco, Ñuble Region
UDEC-LAF 375	I115	*M. importuna*	PZ448489	September 2022	Long-abandoned, disturbed urban gardens, Chillán, Ñuble Region
**UDEC-LAF 292**	**I2**	*M. importuna*	PZ448487	August 2022	Harvested *Pinus radiata* plantation, Santa Fe, Biobío Region
**UDEC-LAF 298**	**I38**	*M. importuna*	PZ448488	August 2022	Harvested *Pinus radiata* plantation, Santa Fe, Biobío Region
UDEC-LAF 322	E62	*M. eximia*	PZ448491	August 2022	Post-fire *Eucalyptus globulus* plantation; Copiulemu, Biobío Region
**UDEC-LAF 336**	**E76**	*M. eximia*	PZ448490	August 2022	Post-fire *Pinus radiata* plantation; Copiulemu, Biobío Region
**UDEC-LAF 347**	**E87**	*M. eximia*	PZ448492	September 2022	Post-fire *Eucalyptus globulus* plantation; Mulchen, Biobío Region
UDEC-LAF 352	E92	*M. eximia*	PZ448493	September 2022	Post-fire *Pinus radiata* plantation; Mulchen, Biobío Region

Strains highlighted in bold correspond to those selected for all in vitro evaluations.

**Table 2:** Semiquantitative evaluation of sclerotia formation in *Morchella* strains cultured under split-plate conditions.

Species/Strain	Abundance	Predominant Size	Coloration	Position
*M. andinensis*–A125	–	–	–	–
*M. andinensis*–A142	++	Large	Yellow	Mixed
*M. tridentina*–T130	–	–	–	–
*M. tridentina*–T140	–	–	–	–
*M. importuna*–I2	++++	Small	White	Submerged
*M. importuna*–I38	+++	Medium	White	Submerged
*M. eximia*–E76	–	–	–	–
*M. eximia*–E87	++	Large	Orange	Superficial

Predominant apparent size was classified as small (<0.5 mm), medium (0.5–1.5 mm), and large (>1.5 mm). Abundance determined as number of sclerotia per plate: – = none; ++ = 6–20; +++ = 21–50; ++++ = >50. Position indicates whether structures were predominantly superficial, submerged in the agar, or present in both positions (mixed). A dash (–) indicates that no sclerotial structures were observed.

## Data Availability

All original data presented in this study are included in the article and in the Supplementary Material. Additional requests can be directed at the corresponding author.

## Supplementary Materials

The following supporting information can be downloaded at: https://www.mdpi.com/article/10.3390/jof12080572/s1, Table S1: Characteristics of the locations where *Morchella* specimens were collected in south-central Chile; Table S2: Replicate counts of structures compatible with sclerotia in *Morchella* strains; Figure S1: Boxplots of radial growth rate and biomass accumulation by strain across carbon sources, nitrogen sources, temperatures, and pH levels; Figure S2: PCA loading plot of radial growth and biomass variables across the evaluated experimental conditions; File S1: Complete statistical analysis report, including descriptive statistics, two-way ANOVA results, model diagnostics, and Tukey’s HSD comparisons; File S2: Molecular data for the *Morchella* specimens, including sequence information, GenBank accession numbers, reference sequences, and sequence-similarity results.
