# Supplementary material for: In Vitro Physiological Characterization of Strains from Transcontinental and Endemic Morchella Species from South-Central Chile
Source: J Fungi (Basel). 2026 Aug 1;12(8):572. doi: 10.3390/jof12080572 (PMC13514834; doi:10.3390/jof12080572)
Supplement: Supplementary file 1 [file jof-12-00572-s001.zip › Supplementary_Material_Tables_and_Figures Elso et al R2.pdf]

Supplementary material

Table S1 Characteristics of the locations where *Morchella* specimens were collected in south-central Chile**.**

Location/Region	Coordinates	Altitude	Predominant Vegetation	Condition
Santa Fe, Biobío Region,	37°29´45.80″S<br>72°35´36.21″ W	95	*P. radiata* (ei)	Harvested forest plantation; soil without plant cover; with abundant branches and debris from pine logging.
Copiulemu, Biobío Region	36°56´39.98″ S<br>72°48´42.61″ W	248	*P. radiata* (ei), *Eucalyptus spp*. (ei)	Forest plantation affected by a recent fire in the summer of 2022; soil without plant cover
Mulchen, Biobío Region	37°44'16.4"S<br>72°13'23.4"W	159	*P. radiata* (ei), *Eucalyptus spp*. (ei)	Forest plantation affected by a recent fire in the summer of 2022; soil without plant cover
Chillan, Ñuble	36°35'12.9"S<br>72°07'51.3"W	122	Mix of shrubs and trees	Old, abandoned urban house with a great diversity of exotic and native vegetation in its gardens, affected by fire in the past.
Villucura, Biobío Region	37°33´28.2″S<br>71°55´12.4″ W	434	*N. obliqua* (d), *L. apiculata* (e)	Conserved relic of the native forest; close proximity to a stream; soil with abundant leaf litter.
Peralillo Biobío Region	37°23´41″ S<br>71°48´27.4″ W	616	*N. obliqua* (d), *N. alpina* (d)	Relatively conserved relic of the native forest surrounded by forest plantations; moderate traffic of people and domestic animals; abundant leaf litter in the soil.
Pemuco, Monte El León 1, Ñuble Region	37°01´14.8″ S<br>71°44´45.1″ W	806	*Nothofagus obliqua* (d), *Luma apiculata* (e), *Gevuina avellana* (e)	Relatively conserved native forest with moderate traffic of people; soil with abundant leaf litter.
Pemuco, Monte El León 2, Ñuble Region	37°01´54.4″ S<br>71°44´59.5″ W	788	*N. obliqua* (d), *Lomatia hirsuta* (e)	Relatively conserved native forest with moderate traffic of people; soil with abundant leaf litter.

Information includes location/administrative region, GPS coordinates, altitude, predominant tree cover, and disturbance type. Vegetation codes: (d) = dominant species, (e) = evergreen, (ei) = exotic/introduced species.

Table S2. Replicate counts of structures compatible with sclerotia in *Morchella* strains

Species/Strain	Plate 1	Plate 2	Plate 3	Mean
M. andinensis - A125	-	-	-	-
M. andinensis - A142	19	7	18	14.67
M. tridentina - T130	-	-	-	-
M. tridentina - T140	-	-	-	-
M. importuna - I2	98	29	27	51.33
M. importuna- I38	49	31	27	35.67
M. eximia - E76	-	-	-	-
M. eximia - E87	20	24	16	20

Number of structures compatible with sclerotia quantified for each *Morchella* strain in three replicate plates. Values correspond to the total number of structures recorded per plate. The mean was calculated from the three replicate plates. A dash (–) indicates that no compatible structures were observed

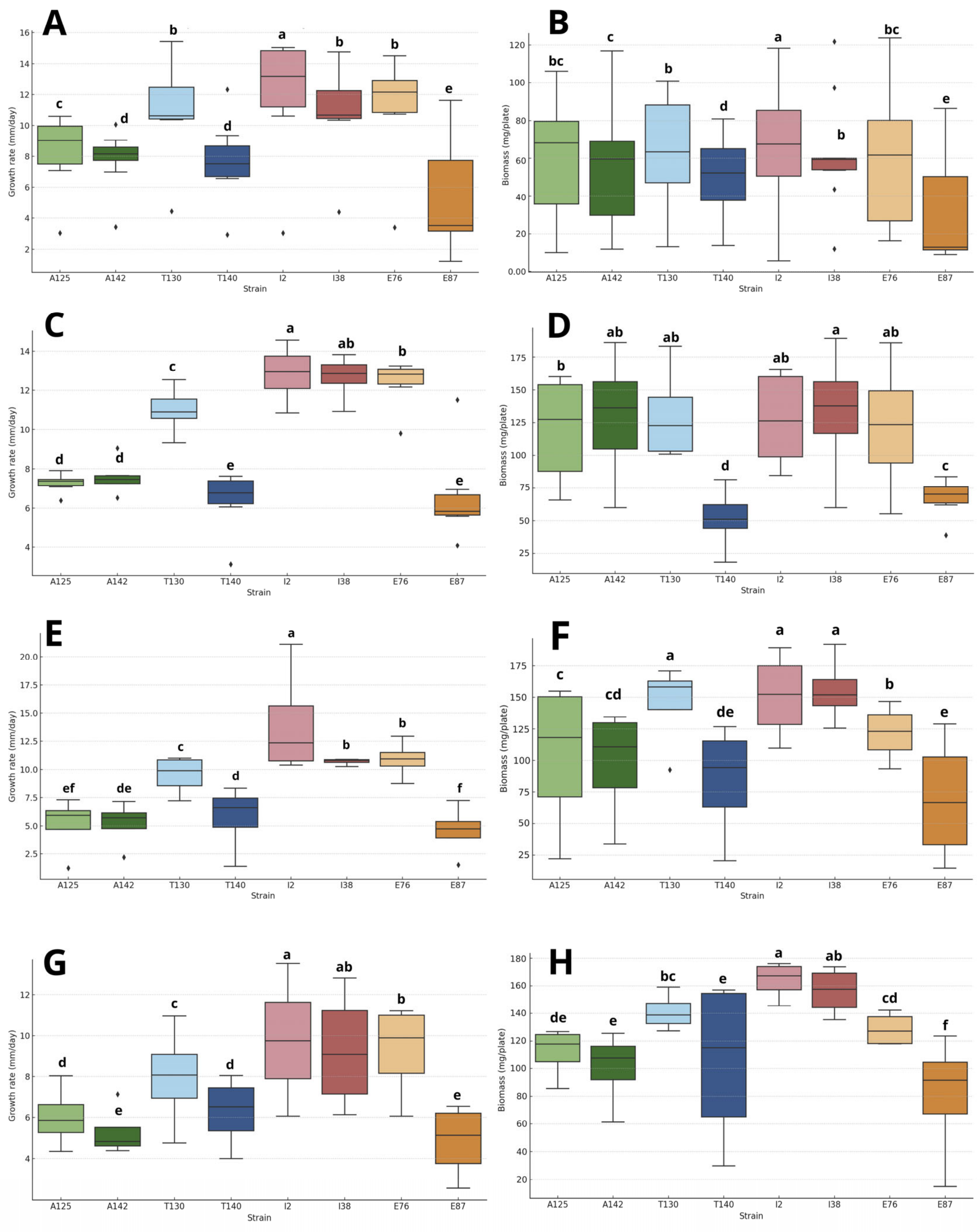

Figure S1. Boxplots of radial growth rate and biomass accumulation by strain across carbon sources, nitrogen sources, temperatures, and pH levels. Boxplots of (A, C, E, G) radial growth rate (mm·day^{-1}) and (B, D, F, H) biomass accumulation (mg·plate^{-1}) by strain across (A, B) carbon sources, (C, D) nitrogen sources, (E, F) temperatures, and (G, H) pH levels. Each box summarizes treatment means (n = 3), showing the median and interquartile range (IQR); whiskers extend to 1.5 × IQR. Points beyond the whiskers are graphical outliers and were retained in the analyses. Letters indicate Tukey's HSD groupings of strain means across the corresponding factor levels ($p < 0.05$); strains sharing a letter do not differ significantly. No letters are shown in panel B because no significant differences were detected. Colors represent *M. andinensis* (green), *M. tridentina* (blue), *M. importuna* (red), and *M. eximia* (yellow).

A

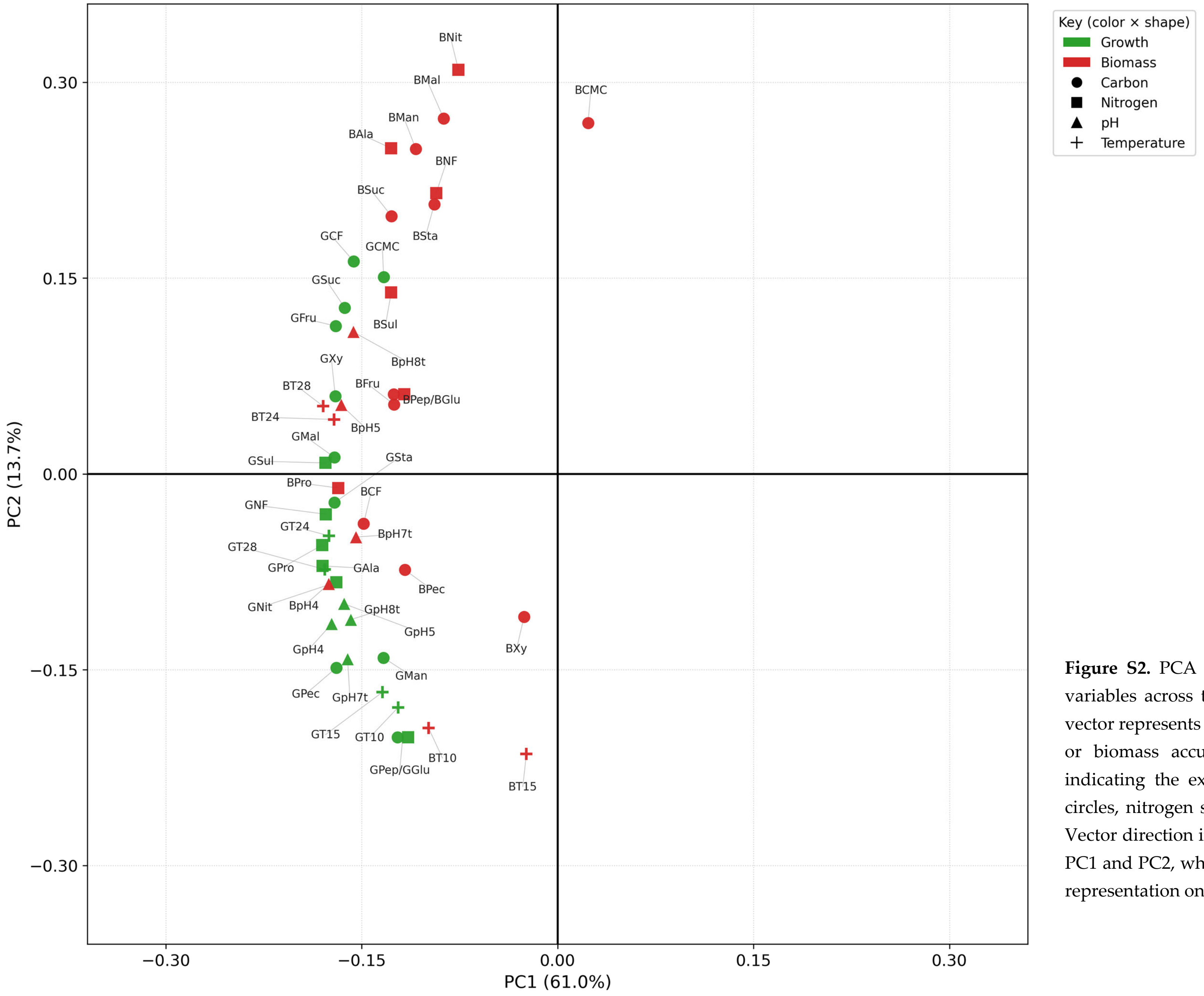

Figure S2. PCA loading plot of radial growth and biomass variables across the evaluated experimental conditions. Each vector represents a single radial growth rate variable (G, green) or biomass accumulation variable (B, red), with symbols indicating the experimental factor (squares, carbon sources; circles, nitrogen sources; triangles, pH; crosses, temperature). Vector direction indicates the association of each variable with PC1 and PC2, whereas vector length reflects the strength of its representation on these two components
